# Hyperkalemia and renin-angiotensin aldosterone system inhibitor therapy in chronic kidney disease: A general practice-based, observational study

**DOI:** 10.1371/journal.pone.0213192

**Published:** 2019-03-07

**Authors:** Min Jun, Meg J. Jardine, Vlado Perkovic, Quentin Pilard, Laurent Billot, Anthony Rodgers, Kris Rogers, Martin Gallagher

**Affiliations:** The George Institute for Global Health, UNSW Sydney, Sydney, Australia; University of Glasgow, UNITED KINGDOM

## Abstract

Data on hyperkalemia frequency among chronic kidney disease (CKD) patients receiving renin-angiotensin aldosterone system inhibitors (RAASis) and its impact on subsequent RAASi treatment are limited. This population-based cohort study sought to assess the incidence of clinically significant hyperkalemia among adult CKD patients who were prescribed a RAASi and the proportion of patients with RAASi medication change after experiencing incident hyperkalemia. We conducted a retrospective, population-based cohort study (1 January 2013–30 June 2017) using Australian national general practice data from the NPS MedicineWise’s MedicineInsight program. The study included adults aged ≥18 years who received ≥1 RAASi prescription during the study period and had CKD (estimated glomerular filtration rate [eGFR] <60 ml/min/1.73m^2^). Study outcomes included incident clinically significant hyperkalemia (serum potassium >6 mmol/L or a record of hyperkalemia diagnosis) and among patients who experienced incident hyperkalemia, the proportion who had RAASi medication changes (cessation or dose reduction during the 210-day period after the incident hyperkalemia event). Among 20,184 CKD patients with a median follow-up of 3.9 years, 1,992 (9.9%) patients experienced an episode of hyperkalemia. The overall incidence rate was 3.1 (95% CI: 2.9–3.2) per 100 person-years. Rates progressively increased with worsening eGFR (e.g. 3.5-fold increase in patients with eGFR <15 vs. 45–59 ml/min/1.73m^2^). Among patients who experienced incident hyperkalemia, 46.6% had changes made to their RAASi treatment regimen following the first occurrence of hyperkalemia (discontinuation: 36.6% and dose reduction: 10.0%). In this analysis of adult RAASi users with CKD, hyperkalemia and subsequent RAASi treatment changes were common. Further assessment of strategies for hyperkalemia management and optimal RAASi use among people with CKD are warranted.

## Introduction

Chronic kidney disease (CKD; defined as estimated glomerular filtration rate [eGFR] <60 ml/min/1.73m^2^ or the presence of markers of kidney damage) is a common global public health threat associated with high morbidity and mortality. Blood pressure lowering therapy has shown to prevent the onset of poor cardiovascular outcomes (which remains the leading of cause of death in CKD) and delay the progression of kidney disease [1,2]. Inhibition of the renin-angiotensin aldosterone system (RAAS) is recommended as first-line blood pressure lowering therapy in CKD based on trials showing specific benefit, and is thus a core component of the management of patients with CKD. However, treatment with RAAS inhibitors (RAASis) including angiotensin-converting enzyme inhibitors (ACEi) and angiotensin II receptor blockers (ARBs) is associated with an increased risk of hyperkalemia [3] (typically defined as serum potassium >5.5 or >6 mmol/L) and this risk can be further exacerbated when used in combination (almost 3-fold compared with RAASi monotherapy)[4].

The clinical implications of the increased risk of RAASi-associated hyperkalemia may be heightened among patients with CKD in whom disturbances in potassium homeostasis are already prevalent [5,6], predisposing this high-risk patient group to hyperkalemia and subsequent adverse outcomes including cardiovascular events. However, studies which have assessed the incidence of hyperkalemia specifically in those with CKD receiving RAASi treatment (i.e. the patient group in whom both the risk of hyperkalemia and the relative risk reduction of adverse outcomes from RAASi therapy may be greatest) and the extent to which hyperkalemia affects subsequent RAASi treatment regimen, have been limited. Recent population-based studies are limited by the inclusion of relatively small proportions of people with CKD [7] or according to level of kidney function, [8] assessment of a cohort which included predominantly males (96%) [9] and/or small size (n = 238 [10] and 258 [11] patients).

We conducted a population-based cohort study of adults CKD patients who were prescribed a RAASi to determine the incidence of clinically significant hyperkalemia, the proportion of patients with RAASi medication change after experiencing incident hyperkalemia and the patient characteristics associated with these outcomes.

## Materials and methods

### Study design and source population

We conducted a retrospective, population-based cohort study using health care data from MedicineInsight, a national general practice data source. MedicineInsight data include longitudinal, de-identified patient information, prescription medications (date of prescription, medication type, strength, dose and quantity and number of prescription repeats) and clinical data (e.g. comorbidities, laboratory test results and other clinical observations) on patients visiting general practices participating in MedicineInsight from all 6 states and 2 territories in Australia. Further details on the MedicineInsight data source is provided elsewhere [12].

### Identification of the study cohort

The study included all adults (≥18 years) who received a prescription for a RAASi (defined as angiotensin converting enzyme inhibitors [ACEi], angiotensin II receptor blockers [ARBs], aldosterone antagonists or combinations of these agents with other blood-pressure lowering medications; S1 Table) during the study period (1 January 2013 to 1 June 2017) and had CKD, defined as an estimated glomerular filtration rate (eGFR) <60 ml/min/1.73m^2^ at index date (S1 Fig). Index date (i.e. “baseline”) was defined as the date of the first eligible RAASi prescription during the study period. We excluded patients who 1) were aged ≤17 years at the start of the study period, 2) had elevated potassium levels >6 mmol/L or coded free-text diagnosis of hyperkalemia recorded in the diagnosis fields of the data ≤365 days prior to the index date, 3) had no information regarding RAASi dose at index date, 4) had no qualifying eGFR measurement or 5) had a recorded diagnosis of CKD with no supporting eGFR measurement.

#### Assessment of kidney function

Eligible patients were those with ≥1 serum creatinine measurement during the period ≤180 days before and ≤180 days after the index date. We estimated baseline eGFR using the Chronic Kidney Disease Epidemiology Collaboration (CKD-EPI) equation [13]. Patients were categorized into the Kidney Disease: Improving Global Outcomes (KDIGO) CKD eGFR categories [14]: 45–59, 30–44, 15–29 and <15 ml/min/1.73m^2^. We excluded participants with end-stage kidney disease (ESKD), defined as having received chronic dialysis or kidney transplantation at baseline.

### Covariates

We obtained information on patient sociodemographic characteristics (sex, age, Indigenous status, Accessibility and Remoteness Index of Australia (ARIA+) [15] [major cities, inner regional, outer regional, remote and very remote], state [Western Australia, Northern Territory, Queensland, South Australia, New South Wales, Australian Capital Territory, Victoria and Tasmania], Socio-Economic Indexes for Areas [SEIFA [16]; from 1 (most disadvantaged) to 10 (most advantaged)], veteran status and healthcare card status), smoking status and comorbid conditions (atrial fibrillation, cardiovascular disease, stroke [all sub-types], heart failure, left ventricular hypertrophy and diabetes mellitus [type 1 and 2] from the relevant data tables of the MedicineInsight database within 1 year prior to the index date.

### Outcomes

The study outcomes of interest included: 1) incident clinically significant hyperkalemia defined as serum potassium >6 mmol/L or coded or free-text recorded diagnosis of hyperkalemia and 2) among patients who experienced incident hyperkalemia, the proportion of patients who had RAASi medication changes defined as RAASi medication cessation or dose reduction during the 210-day period (hereinafter referred to as the medication change ascertainment period; prescriptions for RAASis typically cover a 180-day period–an additional 30-day period was added to allow for delays in GP visits) after the date of the incident hyperkalemia event. RAASi medication cessation was defined as no new RAASi prescription during the medication change ascertainment period, while dose reduction was defined as receiving a prescription for a RAASi at reduced dosage during the RAASi medication change ascertainment period compared with the RAASi treatment dose in the ≤210 days prior to the incident hyperkalemia event. Total daily RAASi dose was derived based on RAASi medication strength, the frequency of administration and total days supplied. Patients were followed from their index date until the date of the hyperkalemia event, death or study end (30 June 2017). While information on the year of death is available in the MedicineInsight general practice clinical information system, specific dates of death are not routinely recorded. For death records with no accompanying dates, we therefore assigned the date “June 30” (end of the Australian financial year).

### Statistical analysis

We used Poisson regression to estimate unadjusted and adjusted incidence rates of hyperkalemia (time to the first event; overall and according to eGFR category), accounting for the clustering effect of general practice sites. Rates (and their 95% confidence intervals [CIs]) were expressed per 100 person-years. Adjusted models included sociodemographic information (sex, age, indigenous status, region of residence, SEIFA, veteran status and healthcare card status), smoking status and comorbid conditions (atrial fibrillation, cardiovascular disease, stroke, heart failure, left ventricular hypertrophy and diabetes. Multivariable Cox regression models (accounting for the clustering effect of general practice sites) were constructed to assess risk factors for incident hyperkalemia and estimate hazard ratios (HRs) and their corresponding 95% confidence intervals (CIs).

For the outcome of RAASi medication change, based on a subgroup of patients who experienced an incident hyperkalemia event, we evaluated the proportion of patients in this group who experienced RAASi dose reduction or discontinuation during the medication change ascertainment period.

Based on the subgroup of patients who had an incident hyperkalemia event, we used multilevel logistic regression (accounting for the clustering effect of general practice sites) to determine patient characteristics associated with change in RAASi treatment regimen. Univariate and full multivariable models including all available covariates were constructed.

#### Subgroup and sensitivity analysis

We assessed adjusted incidence rates of hyperkalemia according to baseline characteristics and disease status including: sex (female and male), age (18–39, 40–59, 60–79 and ≥80 years), Indigenous status (non-Indigenous and Indigenous), region of residence in Australia (inner regional, major cities, outer regional and remote or very remote Australia), smoking status (ex-smoker, non-smoker and smoker), atrial fibrillation, cardiovascular disease, diabetes, heart failure, left ventricular hypertrophy and stroke. We also performed sensitivity analyses to confirm the robustness of our findings. We repeated all analyses using an increased medication change ascertainment period of 270 days. We assessed the incidence of hyperkalemia according to the mean value of serum potassium measurements over the duration of follow up (grouped as <2, 2 to <4 and ≥4 measurements per person-year).

A 2-sided p-value <0.05 was considered statistically significant. All analyses were performed using SAS, version 9.4 (SAS Institute Inc) and Stata software (release 11.2, StataCorp, College Station, TX, USA).

## Ethical approval

This study was approved by the Bellberry Human Research Ethics Committee (2017-09-710).

## Results

### Patient characteristics

**Fig 1:**
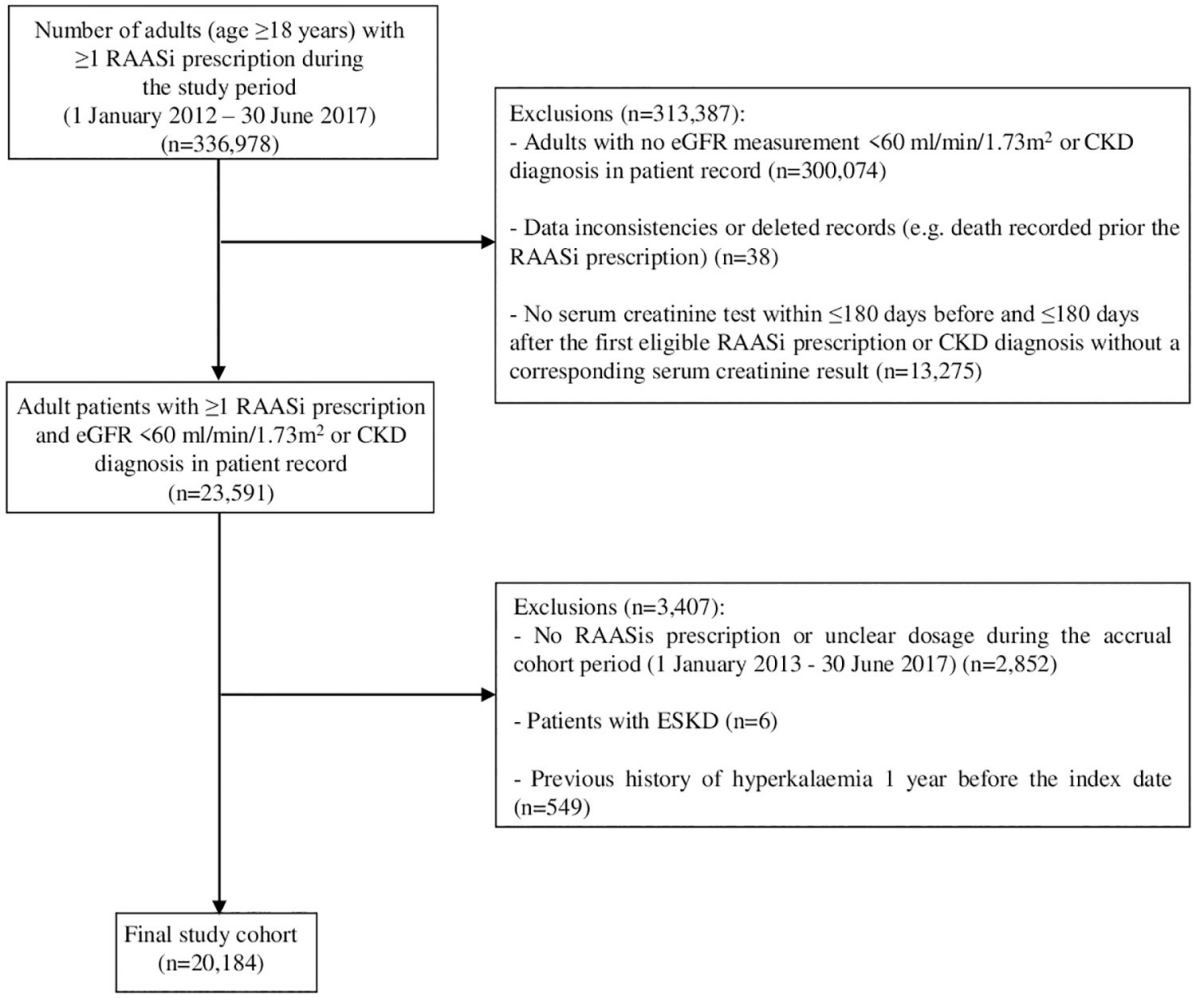
Identification of the study cohort.

We identified 336,978 adult patients with ≥1 RAASi prescription during the study period. Of these patients, 20,184 patients had ≥1 serum creatinine measurements and a corresponding eGFR <60 ml/min/1.73m^2^ and were eligible for inclusion into the study cohort (Fig 1). The mean age of the cohort was 76.9 (SD 9.6) years, 54.5% were female while the mean eGFR was 42.1 ml/min/1.73m^2^ (Table 1). The mean potassium level at baseline was 4.6 (SD 0.5) mmol/L and higher levels were more prevalent among patients with lower levels of eGFR.

**Table 1:** Baseline characteristics of the study cohort, overall and by eGFR category.

	Overall	eGFR category (ml/min/1.73m^2^)			
		45–59	30–44	15–29	<15
	n = 20,184	n = 9,444 (46.8)	n = 7,739 (38.3)	n = 2,632 (13.0)	n = 369 (1.8)
**Sociodemographic information**					
Sex;					
Female	11,009 (54.5)	5,052 (53.5)	4,314 (55.7)	1,468 (55.8)	175 (47.4)
Male	9,175 (45.4)	4,392 (46.5)	3,425 (44.2)	1,164 (44.2)	194 (52.6)
Age, yr, mean, (SD)	76.9 (9.6)	75.8 (9.4)	78.3 (9.0)	78.1 (10.1)	69.7 (14.9)
Age category;					
18–29	26 (0.1)	9 (0.1)	5 (0.1)	5 (0.2)	7 (1.9)
30–39	88 (0.4)	25 (0.3)	32 (0.4)	18 (0.7)	13 (3.5)
40–49	258 (1.3)	113 (1.2)	76 (0.9)	48 (1.8)	21 (5.7)
50–59	733 (3.6)	410 (4.3)	200 (2.6)	85 (3.2)	38 (10.3)
60–69	2,687 (13.3)	1,568 (16.6)	801 (10.3)	255 (9.7)	63 (17.1)
70–79	6,761 (33.5)	3,499 (37.0)	2,412 (31.2)	731 (27.8)	119 (32.2)
≥80	9,631 (47.7)	3,820 (40.4)	4,213 (54.4)	1,490 (56.6)	108 (29.3)
Indigenous status	276/16,078 (1.7)	126/7,653 (1.6)	92/6,077 (1.5)	49/2,048 (2.4)	9/300 (3.0)
ARIA;	n = 20,100	n = 9,399	n = 7,715	n = 2,620	n = 366
Major cities	11,274 (56.1)	5,341 (56.8)	4,278 (55.4)	1,474 (56.3)	181 (49.4)
Inner regional	6,326 (31.5)	2,904/ (30.9)	2,490 (32.3)	798 (30.5)	134 (36.6)
Outer regional	2,250 (11.2)	1,037 (11.0)	864 (11.2)	301 (11.5)	48 (13.1)
Remote	209 (1.0)	97 (1.0)	70 (0.9)	40 (1.5)	2 (0.5)
Very remote	41 (0.2)	20 (0.2)	13 (0.2)	7 (0.3)	1 (0.3)
State;					
Australian Capital Territory	345 (1.7)	186 (2.0)	112 (1.4)	34 (1.3)	13 (3.5)
New South Wales	7,120 (35.3)	3,319 (35.1)	2,709 (35.0)	941 (35.7)	151 (40.9)
Northern Territory	198 (1.0)	76 (0.8)	88 (1.1)	31 (1.2)	3 (0.8)
Queensland	3,097 (15.3)	1,493 (15.8)	1,132 (14.6)	433 (16.4)	39 (10.6)
South Australia	683 (3.4)	305 (3.2)	266 (3.4)	102 (3.9)	10 (2.7)
Tasmania	1,766 (8.7)	828 (8.8)	712 (9.2)	199 (7.6)	27 (7.3)
Victoria	4,711 (23.3)	2,248 (23.8)	1,814 (23.4)	573 (21.8)	76 (20.6)
Western Australia	2,264 (11.2)	989 (10.5)	906 (11.7)	319 (12.1)	50 (13.5)
SEIFA decile;	n = 20,093	n = 9,397	n = 7,711	n = 2,619	n = 366
1 (most disadvantaged)	1,381 (6.9)	650 (6.9)	510 (6.6)	202 (7.7)	19 (5.2)
2	2,970 (14.8)	1,357 (14.4)	1,155 (15.0)	395 (15.1)	63 (17.2)
3	1,698 (8.4)	798 (8.5)	662 (8.6)	212 (8.1)	26 (7.1)
4	1,973 (9.8)	923 (9.8)	761 (9.9)	240 (9.2)	49 (13.4)
5	2,436 (12.1)	1,107 (11.8)	953 (12.4)	339 (12.9)	37 (10.1)
6	2,491 (12.4)	1,161 (12.4)	948 (12.3)	338 (12.9)	44 (12.0)
7	1,465 (7.3)	705 (7.5)	539 (7.0)	188 (7.2)	33 (9.0)
8	1,675 (8.3)	809 (8.6)	635 (8.2)	201 (7.7)	30 (8.2)
9	2,094 (10.4)	997 (10.6)	779 (10.1)	280 (10.7)	38 (10.4)
10 (most advantaged)	1,910 (9.5)	890 (9.5)	769 (1.0)	224 (8.5)	27 (7.4)
Veterans’ status	1,674 (8.3)	659 (7.0%)	731 (9.4)	267 (10.1)	17 (4.6)
Healthcare card status	7,135 (35.5)	3,293 (34.9)	2,807 (36.3)	895 (34.0)	140 (37.9)
Smoking status;	n = 18,851	n = 8.941	n = 7,179	n = 2,404	n = 327
Smoker	872 (4.6)	429 (4.8)	302 (4.2)	118 (4.9)	23 (7.0)
Previous smoker	7,666 (40.7)	3,671 (41.1)	2,882 (40.1)	990 (41.2)	123 (37.6)
Non-smoker	10,313 (54.7)	4,841 (54.1)	3,995 (55.6)	1,296 (53.9)	181 (55.3)
**Laboratory measurements**;					
eGFR (ml/min/1.73m^2^), mean (SD)	42.1 (11.4)	-	-	-	-
Potassium (mmol/L), mean (SD)	4.6 (0.5)	4.5 (0.4)	4.6 (0.5)	4.7 (0.5)	4.7 (0.6)
Potassium category;	n = 17,131	n = 8,028	n = 6,595	n = 2,217	n = 291
<3.5	153 (0.9)	66 (0.8)	50 (0.8)	27 (1.2)	10 (3.4)
3.5–4.9	13,436 (78.4)	6,727 (83.8)	5,030 (76.3)	1,512 (68.2)	167 (57.4)
≥5.0	3,542 (20.7)	1,235 (15.4)	1,515 (23.0)	678 (30.6)	114 (39.2)
**Comorbid conditions**;					
Atrial fibrillation	4,547 (22.5)	2,080 (22.0)	1,776 (22.9)	630 (23.9)	61 (16.5)
Cardiovascular disease	11,215 (55.6)	4,957 (52.5)	4,417 (57.1)	1,645 (62.5)	196 (53.1)
Diabetes mellitus	8,238 (40.8)	3,748 (39.7)	3,074 (39.7)	1,240 (47.1)	176 (47.7)
Heart failure	5,397 (26.7)	2,226 (23.6)	2,148 (27.8)	924 (35.1)	99 (26.8)
Left ventricular hypertrophy	286 (1.4)	158 (1.7)	90 (1.2)	34 (1.3)	4 (1.1)
Stroke	2,683 (13.3)	1,232 (13.0)	1,066 (13.8)	347 (13.2)	38 (10.3)
**RAASi type**;					
ACEi	6,117 (30.1)	2,920 (30.9)	2,309 (29.8)	777 (29.5)	111 (30.1)
ARB	8,336 (41.3)	3,901 (41.3)	3,182 (41.1)	1,076 (40.9)	177 (47.9)
Aldosterone antagonist	1,648 (8.2)	593 (6.3)	699 (9.0)	324 (12.3)	32 (8.7)
ACEi + other combinations	900 (4.4)	475 (5.0)	321 (4.1)	95 (3.6)	9 (2.4)
ARB + other combinations	3,183 (15.8)	1555 (16.5)	1228 (15.9)	360 (13.7)	40 (10.8)

Values are numbers (percentages) unless stated otherwise; For variables with incomplete data availability, the denominators have been provided; SD = standard deviation; ARIA = Accessibility and Remoteness Index of Australia; SEIFA = socio-economic indexes for areas; eGFR = estimated glomerular filtration rate; RAASi = renin-angiotensin aldosterone system inhibitor; ACEi = angiotensin-converting enzyme inhibitor; ARB = angiotensin II receptor blocker

### Incidence of hyperkalemia after RAASi initiation

**Fig 2:**
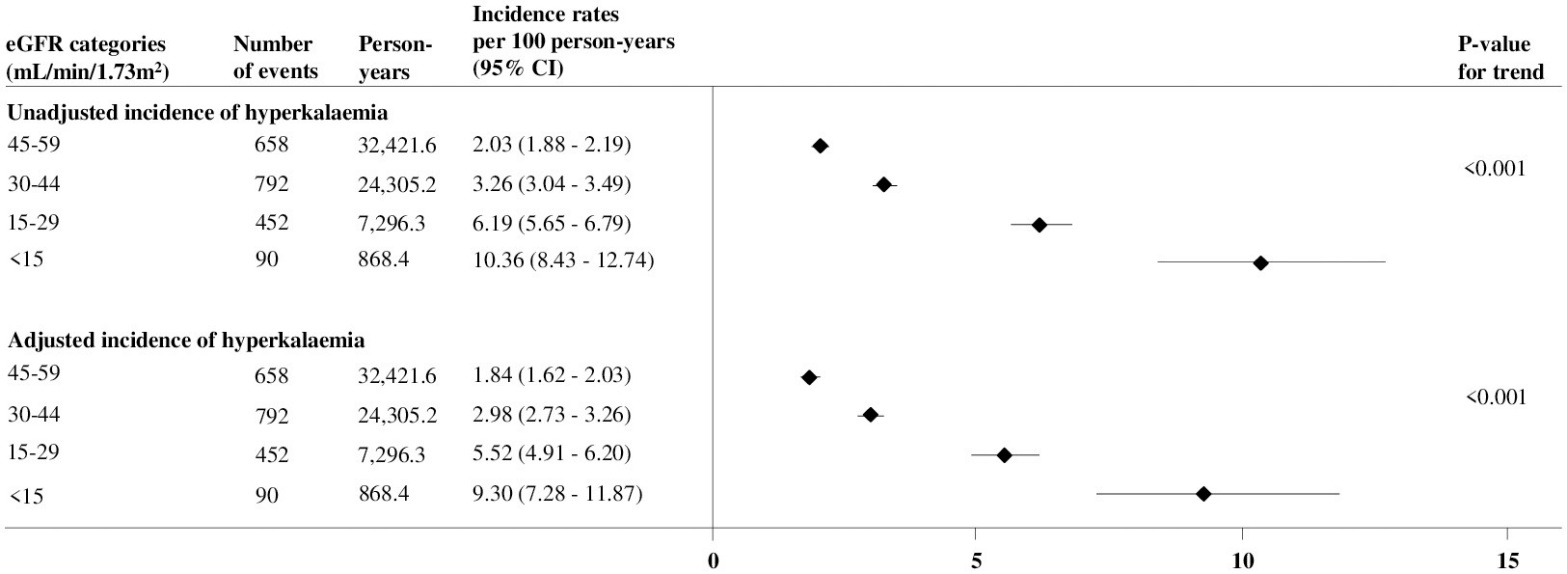
Unadjusted and adjusted* incidence rate of hyperkalemia according to eGFR category.

Over a median follow-up of 3.9 years (interquartile interval 2.2–4.3), 1,992 (9.9%) patients experienced an episode of hyperkalemia. The overall incidence of hyperkalemia was 3.1 (95% CI: 2.9–3.2) per 100 person-years. Overall, incidence of hyperkalemia progressively increased with worsening levels of eGFR (p for trend<0.001; Fig 2). The adjusted rate of hyperkalemia among those with eGFR <15 ml/min/1.73m^2^ was 3.5-fold higher compared with patients with eGFR 45–59 ml/min/1.73m^2^. Overall, mortality during the study period was higher among patients who experienced incident hyperkalemia (356/1,992; 17.9%) compared with those who did not (2,051/18,192; 11.3%).

In multivariable Cox regression models, female sex (vs. male: HR 0.76, 95% CI: 0.67–0.86) and older age (per 1 year increase: HR 0.99, 95% CI: 0.98–0.99) were associated with a lower risk of incident hyperkalemia (Table 2). Patient characteristics associated with significantly increased hyperkalemia risk included smoking status (e.g. smoker vs. non-smoker: HR 1.38, 95% CI: 1.05–1.80), worsening eGFR level (e.g. eGFR 45–59 vs. <15 ml/min/1.73m^2^: HR 3.73, 95% CI: 2.71–5.13), baseline serum potassium level (per 0.1 mmol/L increase: HR 1.13, 95% CI: 1.12–1.14), diabetes (yes vs. no: HR 1.26, 95% CI: 1.12–1.41) and heart failure (yes vs. no: HR 1.38, 95% CI: 1.19–1.60).

**Table 2:** Patient characteristics associated with incident hyperkalemia among patients with CKD.

	Univariate model; HR (95% CI)	P-value	Full multivariable model; HR (95% CI)	P-value
**Sociodemographic information**				
Sex (female vs male)	0.68 (0.61–0.73)	<0.001	0.76 (0.67–0.86)	<0.001
Age (per 1 year increase)	0.99 (0.99–0.99)	0.032	0.99 (0.98–0.99)	0.044
Indigenous status (Indigenous vs non-Indigenous)	1.26 (0.90–1.77)	0.183	1.16 (0.76–1.76)	0.484
ARIA;				
Major cities (reference)	1.00	0.558	1.00	0.855
Inner regional	0.79 (0.55–1.13)		0.86 (0.54–1.36)	
Outer regional	0.80 (0.49–1.32)		0.90 (0.48–1.69)	
Remote	0.39 (0.10–1.53)		0.34 (0.04–3.13)	
Very remote	0.62 (0.08–4.70)		0.62 (0.07–5.10)	
SEIFA decile;				
1 (most disadvantaged)	0.96 (0.66–1.40)	0.185	0.91 (0.55–1.50)	0.419
2	1.16 (0.81–1.65)		0.94 (0.57–1.54)	
3	0.87 (0.61–1.23)		0.83 (0.51–1.34)	
4	0.76 (0.53–1.08)		0.68 (0.42–1.10)	
5	1.10 (0.79–1.53)		1.08 (0.68–1.71)	
6	0.89 (0.64–1.23)		0.93 (0.59–1.45)	
7	0.93 (0.67–1.29)		0.99 (0.63–1.54)	
8	1.04 (0.76–1.43)		1.12 (0.72–1.72)	
9	0.93 (0.72–1.21)		0.88 (0.62–1.26)	
10 (most advantaged; reference)	1.00		1.00	
Veterans’ status (veteran vs non-veteran)	1.00 (0.85–1.18)	0.978	0.86 (0.67–1.10)	0.226
Healthcare card status (holder vs non-holder)	0.91 (0.79–1.06)	0.237	0.86 (0.70–1.06)	0.171
Smoking status;				
Non-smoker (reference)	1.00	<0.001	1.00	0.025
Previous smoker	1.31 (1.19–1.44)		1.12 (0.99–1.27)	
Smoker	1.55 (1.26–1.91)		1.38 (1.05–1.80)	
**Laboratory measurements**;				
eGFR category;				
45–59 (reference)	1.00	<0.001	1.00	<0.001
30–44	1.60 (1.44–1.77)		1.42 (1.25–1.63)	
15–29	3.08 (2.72–3.48)		2.45 (2.09–2.86)	
<15	5.14 (4.09–6.46)		3.73 (2.71–5.13)	
Serum potassium (per 0.1 mmol/L increase)*	1.14 (1.13–1.15)	<0.001	1.13 (1.12–1.14)	<0.001
**Comorbid conditions**;				
Atrial fibrillation (yes vs no)	1.22 (1.11–1.36)	<0.001	1.10 (0.96–1.26)	0.183
Cardiovascular disease (yes vs no)	1.60 (1.45–1.76)	<0.001	1.12 (0.96–1.30)	0.153
Diabetes (yes vs no)	1.62 (1.48–1.77)	<0.001	1.26 (1.12–1.41)	<0.001
Heart failure (yes vs no)	1.70 (1.55–1.87)	<0.001	1.38 (1.19–1.60)	<0.001
Left ventricular hypertrophy (yes vs no)	0.99 (0.69–1.42)	0.962	0.86 (0.54–1.36)	0.520
Stroke (yes vs no)	0.99 (0.86–1.13)	0.855	0.93 (0.78–1.10)	0.407
**RAASi type**;				
ACEi (reference)	1.00	<0.001	1.00	<0.001
ARB	0.81 (0.72–0.90)		0.95 (0.83–1.09)	
Aldosterone antagonist	1.50 (1.29–1.75)		1.53 (1.25–1.87)	
ACEi + other combinations	1.00 (0.81–1.24)		1.20 (0.93–1.57)	
ARB + other combinations	0.61 (0.52–0.70)		0.81 (0.67–0.98)	

ARIA = Accessibility and Remoteness Index of Australia; HR = hazard ratio; CI = confidence interval; SEIFA = socio-economic indexes for areas; eGFR = estimated glomerular filtration rate;

*Serum potassium level at baseline

### RAASi medication changes after incident hyperkalemia

Of the 1,992 patients who experienced incident hyperkalemia, 1,740 patients (87.3%) had received a prescription for a RAASi ≤210 days prior to the incident hyperkalemia event. Among these patients, 811 (46.6%) had changes made to their RAASi treatment regimen during the period ≤210 days after the first occurrence of hyperkalemia (637 [36.6%] discontinued RAASi treatment and 174 [10.0%] were prescribed a reduced dose of these agents). No significant difference in mean serum potassium levels at the time of incident hyperkalemia according to the type of RAASi medication change (discontinuation or dose reduction; 6.3 [SD 0.4] and 6.2 [SD 0.3] mmol/L, respectively; p for difference = 0.06) was observed. When assessed according to eGFR category, RAASi medication changes were more prevalent among those with lower levels of eGFR (S2 Table).

On univariate analysis, medication change was predicted by healthcare card status (OR 0.77, 95% CI: 0.63–0.94; Table 3) and higher levels of serum potassium at the time of the hyperkalemia event (per 0.1 mmol/L increase: OR 1.04, 95% CI: 1.01–1.08). In the full multivariable model, only higher levels of serum potassium at the time of the hyperkalemia event remained significantly associated with a greater likelihood of medication change (per 0.1 mmol/L increase: OR 1.05, 95% CI: 1.02–1.08).

**Table 3:** Patient characteristics associated with RAASi medication change following incident hyperkalemia among patients with CKD.

	Univariate model; OR (95% CI)	P-value	Full multivariable model; OR (95% CI)	P-value
**Sociodemographic information**				
Sex (female vs male)	0.96 (0.80–1.15)	0.670	1.04 (0.83–1.31)	0.732
Age (per 1 year increase)	1.00 (0.99–1.01)	0.961	1.00 (0.99–1.01)	0.565
Indigenous status (Indigenous vs non-Indigenous)	1.34 (0.70–2.55)	0.379	1.24 (0.62–2.52)	0.548
ARIA;				
Major cities (reference)	1.00	0.562^α^	1.00	0.724^β^
Inner regional	1.12 (0.90–1.39)		1.12 (0.85–1.47)	
Outer regional	1.14 (0.85–1.52)		1.20 (0.84–1.73)	
Remote or very remote^	0.75 (0.33–1.74)		0.88 (0.36–2.15)	
SEIFA decile;				
1 (most disadvantaged)	0.68 (0.44–1.08)	0.173^α^	0.55 (0.31–0.97)	0.196^β^
2	0.90 (0.62–1.31)		0.74 (0.44–1.23)	
3	1.00 (0.65–1.54)		0.76 (0.45–1.31)	
4	0.68 (0.43–1.07)		0.54 (0.31–0.94)	
5	0.70 (0.47–1.06)		0.49 (0.29–0.83)	
6	0.83 (0.55–1.25)		0.66 (0.39–1.11)	
7	0.67 (0.41–1.09)		0.55 (0.30–0.99)	
8	1.12 (0.72–1.75)		0.73 (0.42–1.27)	
9	0.73 (0.48–1.11)		0.60 (0.35–1.03)	
10 (most advantaged; reference)	1.00		1.00	
Veterans’ status (veteran vs non-veteran)	1.40 (1.00–1.95)	0.048	1.25 (0.81–1.94)	0.306
Healthcare card status (holder vs non-holder)	0.77 (0.63–0.94)	0.009	0.85 (0.67–1.08)	0.192
Smoking status;				
Non-smoker (reference)	1.00	0.506^α^	1.00	0.242^β^
Previous smoker	1.04 (0.86–1.26)		1.06 (0.84–1.33)	
Smoker	0.81 (0.52–1.24)		0.68 (0.41–1.13)	
**Laboratory measurements**;				
eGFR category;				
45–59 (reference)	1.00	0.173^α^	1.00	0.654^β^
30–44	1.22 (0.95–1.56)		1.19 (0.88–1.59)	
15–29	1.01 (0.82–1.26)		1.05 (0.81–1.35)	
<15	1.44 (0.92–2.26)		1.23 (0.71–2.12)	
Serum potassium (per 0.1 mmol/L increase)*	1.04 (1.01–1.08)	0.016	1.05 (1.02–1.08)	0.002
**Comorbid conditions**;				
Atrial fibrillation (yes vs no)	1.12 (0.91–1.37)	0.299	1.17 (0.90–1.53)	0.229
Cardiovascular disease (yes vs no)	1.05 (0.86–1.27)	0.644	0.90 (0.67–1.21)	0.482
Diabetes (yes vs no)	0.97 (0.81–1.16)	0.713	1.01 (0.81–1.26)	0.907
Heart failure (yes vs no)	1.12 (0.93–1.36)	0.230	1.20 (0.91–1.59)	0.203
Left ventricular hypertrophy (yes vs no)	1.64 (0.80–3.36)	0.175	1.75 (0.77–3.97)	0.184
Stroke (yes vs no)	1.01 (0.77–1.32)	0.946	1.00 (0.70–1.41)	0.643

OR = odds ratio; CI = confidence interval; ARIA = Accessibility and Remoteness Index of Australia; SEIFA = socio-economic indexes for areas; eGFR = estimated glomerular filtration rate;

^α^Global p-value testing for difference in ORs across the categorical variable in the univariate model;

^β^Global p-value testing for difference in ORs across the categorical variable in the multivariable model;

^Due to small numbers in the “very remote” group, this group was combined with the “remote” group;

*Serum potassium level at the time of the hyperkalemia event

### Subgroup and sensitivity analysis

**Fig 3:**
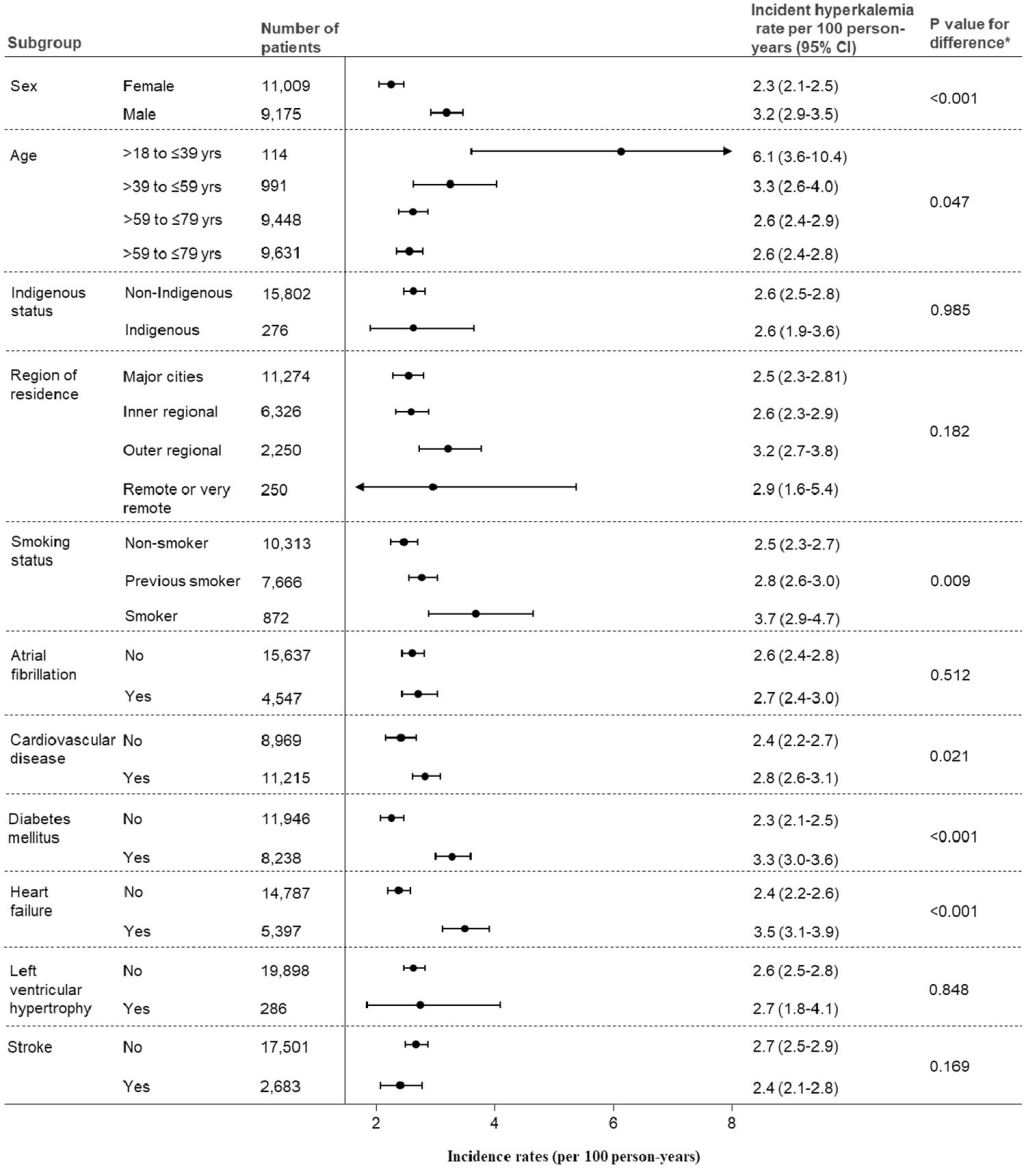
Subgroup analysis assessing hyperkalemia incidence according to baseline patient characteristics.

We observed significant differences in hyperkalemia incidence rates according to age (lower incidence rate with older age; e.g. >18 to ≤39 years vs. >80 years: 6.1 [95% CI: 3.6–10.4] and 2.6 [95% CI: 2.4–2.8] per 100 person-years, respectively; p = 0.047), smoking status (higher incidence rate among smokers; e.g. smoker vs. non-smoker: 3.7 [95% CI: 2.9–4.7] and 2.5 [95% CI: 2.3–2.7] per 100 person-years; p = 0.009) and by the presence of comorbid conditions including cardiovascular disease (yes vs. no: 2.8 [95% CI: 2.6–3.1] and 2.4 [95% CI: 2.2–27] per 100 person-years, respectively; p = 0.021), diabetes (yes vs. no: 3.3 [95% CI: 3.0–3.6] and 2.3 [95% CI: 2.1–2.5] per 100 person-years; p<0.001) and heart failure (yes vs. no: 3.5 [95% CI: 3.1–3.9] and 2.4 [95% CI: 2.2–2.6] 100 person-years; p<0.001; Fig 3). Hyperkalemia incidence rate among people with both diabetes and heart failure (comorbid conditions which each remained significant predictors of hyperkalemia in multivariable Cox models; Table 2) was 3.7 (95% CI: 3.2–4.2) per 100 person-years. Overall, similar results were observed when analyses were repeated 1) using a baseline CKD ascertainment period defined as 1 year prior to or 90 days after the index date (S2 Fig) and 2) using an increased medication change ascertainment period (270 days; S3 Fig; S3 Table). The incidence of hyperkalemia was higher among those with more frequent serum potassium measurements (S4 Fig).

## Discussion

In this population-based cohort study of over 20,000 adult RAASi users with CKD, we observed an overall hyperkalemia incidence of 3.1 per 100 person-years which progressively increased with worsening levels of kidney function. The incidence of hyperkalemia was 3.5-fold higher among patients with eGFR <15 ml/min/1.73m^2^ compared with patients with eGFR 45–59 ml/min/1.73m^2^. Overall, among patients who experienced incident hyperkalemia, 47% had changes made to their RAASi treatment regimen following the event which primarily included RAASi medication cessation. These finding suggest that hyperkalemia may be limiting optimal RAASi utilisation in people with CKD.

A systematic review of 39 randomized trials assessing the effects of RAAS inhibitors and other blood pressure lowering agents in CKD reported that the overall incidence of hyperkalemia (serum potassium ≥6 mmol/L) with single or dual RAAS inhibition generally ranged between 1.9 to 6.1% over 3 months to 3.4 years [3]. A small number of population-based studies on the assessment of the incidence of hyperkalemia across several patient groups have reported highly variable rates. An analysis of the U.S. Veterans Health Administration data (n = 245,808) reported a hyperkalemia incidence (defined as serum potassium ≥6 mEq/L) of 2.6 per 100 person-months (approximately equal to 31.2 per 100 person-years) in a subgroup of RAASi users with CKD [9]. Three other recent population-based cohort studies (each conducted in the U.S. [n = 194,456 [17]], Sweden [n = 69,426 [7]] and Denmark [n = 157,766 [18]]) have reported hyperkalemia incidence (defined as serum potassium >5.0 or >5.5 mEq/L) in CKD (eGFR <60 ml/min/1.73m^2^) or subgroups of patients with CKD from a broader population. These studies have reported an incidence of hyperkalemia in CKD of 4.1% over 3 years [17], 45% over 1 year [7] and 7 per 100 person-years [18], respectively. However, these studies have been limited by a relatively short follow-up period (e.g. <1 year) [9,17] and lack of a nationally representative study sample [7,19].

The variable rates of hyperkalemia across recent studies may be attributable to differences in study inclusion criteria (broad [e.g. age ≥18 years with blood pressure measurement] or specific [e.g. RAAS inhibitor users]) or definitions of hyperkalemia (e.g. serum potassium >5, >5.5 or >6 mmol/L). Nevertheless, collectively, they suggest that hyperkalemia rates derived from randomized trials may not reflect actual incidence in routine clinical practice (as indicated by the generally higher hyperkalemia rates reported by population-based observational studies.

Our study, using a large, national cohort of RAASi users with CKD and a study follow-up of 4.5 years, also shows that hyperkalemia is observed at progressively higher rates among those with poorer levels of kidney function, as well as those with diabetes and heart failure, a finding that is consistent with prior studies reporting increased risk of hyperkalemia among individuals with these conditions. Given that diabetes and heart failure are observed at greater frequency among people with advanced CKD, the complex interplay of these conditions might explain the higher rates of hyperkalemia observed in those with lower levels of eGFR (30–44, 15–29 and <15 ml/min/1.73m^2^) in our study. Similar findings were reported in a recent U.S.-based study [19].

Reported percentages of RAASi therapy (single or dual) discontinuation due to hyperkalemia from randomized trials of various patient groups (e.g. hypertension or heart failure) have been relatively low (0.4–8.1% [3,20]) while data on the patterns of RAASi therapy following hyperkalemia in CKD in routine clinical settings are limited. One population-based study reported a RAASi discontinuation or dose reduction prevalence of 35.2% and 1.9% [17], respectively, among patients with eGFR <30 ml/min/1.73m^2^ (n = 346) while another study reported that among patients who received RAASi therapy at maximum dosage, 47% had their dose reduced or therapy discontinued following a hyperkalemia event (defined as ≥5.5 mEq/L) [21]. Our results are consistent with these data and suggest that RAASi medication change is relatively common in routine clinical settings among CKD patients who experience hyperkalemia. Such changes in RAASi treatment regimens following an episode of hyperkalemia may have important consequences on the trajectory of future cardiovascular and renal risk.

We observed that the incidence of hyperkalemia was higher among those in whom serum potassium was measured more frequently. Patient data (e.g. serum potassium results) from non-MedicineInsight general practices were not available for analysis and while it is possible that such data may have affected serum potassium testing frequency in the current study, it is reasonable to expect that closer monitoring of potassium levels would lead to greater detection of clinically significant hyperkalemia (and vice versa) which in turn may lead to improved management of RAASi treatment regimens. Indeed, we observed that serum potassium levels at the time of the hyperkalaemia event was the strongest predictor of RAASi medication change. Closer patient monitoring attributed to older age and higher comorbid burden may also explain our findings which showed a lower risk of hyperkalemia with older age, results which are consistent with 3 studies assessing the relationship between antihypertensive agents and the risk of hyperkalemia among individuals with varying degrees of kidney function (n = 238 to 55,266 [10,17,19]). Taken together, our data highlights the potential importance of identifying optimal patient monitoring strategies including serum potassium testing in the management of hyperkalemia in this high-risk population.

Our study was based on a large, nationally representative (inclusive of various geographic regions [urban and rural] as well as all states and territories of Australia), population-based cohort of patients with CKD reflecting real-world clinical settings. In addition, we assessed the incidence of hyperkalemia in RAASi users with CKD based on longitudinal data, which allowed for assessments of subsequent medication changes and factors associated with this change. However, our study has some limitations that should be considered. General practices participating in the MedicineInsight program were recruited using non-random sampling. Therefore, while the overall distribution of patients in our study cohort by state and territory was largely consistent with the broader Australian population distribution, sampling differences across Australian regions, states and territories cannot be excluded. There are also some limitations to the data that should be noted including: 1) the potential for incomplete data given that certain variables are not collected within the MedicineInsight program to preserve patient confidentiality, 2) the lack of specific dates pertaining to birth and death (year of birth and death are available) and 3) the potential for duplication of patients across MedicineInsight participating general practices (although, given the general practice setting, we do not expect significant levels of patient movement across practices). We defined a hyperkalemia event as potassium >6 mmol/L to avoid spurious elevations of potassium. Nevertheless, we were limited in our ability to confirm actual levels of potassium in those with known cases of spurious potassium elevations.

We were also limited in our ability to assess for the use of concomitant medications including those known to increase potassium concentrations such as calcineurin inhibitors, beta-blockers and trimethoprim. We sought to delineate descriptively the relationship between RAASi use, kidney function and study outcomes specifically in a population likely to benefit most from continued RAASi therapy (i.e. CKD). Accordingly, we did not include a “control” group of individuals with normal kidney function and/or receiving other blood pressure lowering medications to whom the risk of study outcomes could be compared against. Finally, we did not have complete information on the receipt of chronic dialysis or kidney transplantation at baseline and the identification of the prevalence of these treatments was limited to relevant text searches of the datasets. Thus, the potential for inclusion of patients who received chronic dialysis or kidney transplantation cannot be excluded.

In conclusion, in this study of a large cohort of adults RAASi users with CKD, the incidence of hyperkalemia was frequently observed and rates increased with deteriorating levels of kidney function. RAASi medication change following an episode of hyperkalemia occurred in almost half of patients who experienced hyperkalemia.

## Supporting information

S1 Table RAASi medications and relevant ATC codes. (DOCX)

S2 Table Sensitivity analysis assessing the proportion of patients who had RAASi medication changes according to eGFR category. (DOCX)

S3 Table Sensitivity analysis on the assessment of patient characteristics associated with RAASi medication change following incident hyperkalemia based on an increased medication change ascertainment period (270 days). (DOCX)

S1 Fig Overview of the study design. (DOCX)

S2 Fig Sensitivity analysis using a baseline CKD ascertainment period defined as 1 year prior to or 90 days after the index date. (DOCX)

S3 Fig Sensitivity analysis assessing RAASi medication change using an increased medication change ascertainment period (270 days). (DOCX)

S4 Fig Unadjusted incidence rate of hyperkalemia according to serum potassium testing frequency and eGFR category. (DOCX)
