# Supplementary material for: Hyperkalemia and renin-angiotensin aldosterone system inhibitor therapy in chronic kidney disease: A general practice-based, observational study
Source: PLoS One. 2019 Mar 7;14(3):e0213192. doi: 10.1371/journal.pone.0213192 (PMC6405190; doi:10.1371/journal.pone.0213192)
Supplement: S1 Table — (DOCX) [file pone.0213192.s001.docx]

**Supporting information**

**S1 Table: RAASi medications and relevant ATC codes**

| **RAASi agent** | **Drug name** | **ATC codes** |
| --- | --- | --- |
| ACEi | Captopril | C09AA01 |
|  | Enalapril | C09AA02 |
|  | Fosinopril | C09AA09 |
|  | Lisinopril | C09AA03 |
|  | Perindopril | C09AA04 |
|  | Quinapril | C09AA06 |
|  | Ramipril | C09AA05 |
|  | Trandolapril | C09AA10 |
| ACEi + CCB | Enalapril and lercanidipine | C09BB02 |
|  | Perindopril and amlodipine | C09BB04 |
|  | Ramipril and felodipine | C09BB05 |
|  | Trandolapril and verapamil | C09BB10 |
| ACEi + diuretic | Enalapril and diuretics | C09BA02 |
|  | Fosinopril and diuretics | C09BA09 |
|  | Perindopril and diuretics | C09BA04 |
|  | Quinapril and diuretics | C09BA06 |
| ARB | Candesartan | C09CA06 |
|  | Eprosartan | C09CA02 |
|  | Irbesartan | C09CA04 |
|  | Losartan | C09CA01 |
|  | Olmesartan | C09CA08 |
|  | Telmisartan | C09CA07 |
|  | Valsartan | C09CA03 |
| ARB + CCB | Olmesartan medoxomil and amlodipine | C09DB02 |
|  | Telmisartan and amlodipine | C09DB04 |
|  | Valsartan and amlodipine | C09DB01 |
| ARB + diuretic | Candesartan and diuretics | C09DA06 |
|  | Eprosartan and diuretics | C09DA02 |
|  | Irbesartan and diuretics | C09DA04 |
|  | Olmesartan medoxomil and diuretics | C09DA08 |
|  | Telmisartan and diuretics | C09DA07 |
|  | Valsartan and diuretics | C09DA03 |
| ARB + other combination | Olmesartan medoxomil, amlodipine and hydrochlorothiazide | C09DX03 |
|  | Valsartan, amlodipine and hydrochlorothiazide | C09DX01 |
|  | Valsartan and sacubitril | C09DX04 |
| AA | Eplerenone | C03DA04 |
|  | Spironolactone | C03DA01 |

RAASi=renin-angiotensin aldosterone system inhibitor; ATC=anatomical therapeutic chemical; ACEi=angiotensin converting enzyme inhibitor; CCB=calcium channel blocker; ARB=angiotensin II receptor blocker; AA=aldosterone antagonist
