# Supplementary material for: Hyperkalemia and renin-angiotensin aldosterone system inhibitor therapy in chronic kidney disease: A general practice-based, observational study
Source: PLoS One. 2019 Mar 7;14(3):e0213192. doi: 10.1371/journal.pone.0213192 (PMC6405190; doi:10.1371/journal.pone.0213192)
Supplement: S2 Table — (DOCX) [file pone.0213192.s002.docx]

**Supporting information**

**S2 Table: Sensitivity analysis assessing the proportion of patients who had RAASi medication changes according to eGFR category**

| **eGFR ml/min/1.73m^2^** | **Patients with no new RAASi prescription or RAASi dose reduction** | **Patients with no new RAASi prescription** | **Patients with RAASi dose reduction** |
| --- | --- | --- | --- |
| 45-59 | 257 (43.7%) | 194 (33.0%) | 63 (10.7%) |
| 30-44 | 312 (45.5%) | 239 (34.8%) | 73 (10.6%) |
| 15-29 | 199 (50.8%) | 165 (42.1%) | 34 (8.7%) |
| ˂15 | 43 (58.1%) | 39 (52.7%) | 4 (5.4%) |
