# Supplementary material for: Hyperkalemia and renin-angiotensin aldosterone system inhibitor therapy in chronic kidney disease: A general practice-based, observational study
Source: PLoS One. 2019 Mar 7;14(3):e0213192. doi: 10.1371/journal.pone.0213192 (PMC6405190; doi:10.1371/journal.pone.0213192)
Supplement: S3 Table — (DOCX) [file pone.0213192.s003.docx]

**Supporting information**

**S3 Table: Sensitivity analysis on the assessment of patient characteristics associated with RAASi medication change following incident hyperkalemia based on an increased medication change ascertainment period (270 days)**

|  | **Univariate model; OR (95% CI)** | **P-value** | **Full multivariable model; OR (95% CI)** | **P-value** |
| --- | --- | --- | --- | --- |
| **Sociodemographic information** |  |  |  |  |
| Sex (female vs male) | 0.97 (0.81-1.17) | 0.765 | 1.03 (0.82-1.30) | 0.774 |
| Age (per 1 year increase) | 1.00 (0.99-1.01) | 0.462 | 1.00 (0.99-1.01) | 0.993 |
| Indigenous status (Indigenous vs non-Indigenous) | 1.18 (0.62-2.26) | 0.607 | 1.23 (0.60-2.51) | 0.568 |
| ARIA; |  |  |  |  |
| Major cities (reference) | 1.00 | 0.415^α^ | 1.00 | 0.652^β^ |
| Inner regional | 1.15 (0.94-1.40) |  | 1.11 (0.84-1.47) |  |
| Outer regional | 1.19 (0.90-1.56) |  | 1.25 (0.88-1.78) |  |
| Remote or very remote^ | 0.86 (0.38-1.94) |  | 0.98 (0.41-2.37) |  |
| SEIFA decile; |  |  |  |  |
| 1 (most disadvantaged) | 0.65 (0.41-1.01) | 0.239^α^ | 0.57 (0.32-1.00) | 0.275^β^ |
| 2 | 0.89 (0.62-1.28) |  | 0.75 (0.45-1.25) |  |
| 3 | 0.92 (0.61-1.39) |  | 0.76 (0.44-1.29) |  |
| 4 | 0.71 (0.46-1.09) |  | 0.59 (0.34-1.02) |  |
| 5 | 0.68 (0.46-1.01) |  | 0.51 (0.30-0.85) |  |
| 6 | 0.77 (0.52-1.15) |  | 0.69 (0.42-1.15) |  |
| 7 | 0.61 (0.37-0.99) |  | 0.52 (0.29-0.95) |  |
| 8 | 0.95 (0.61-1.46) |  | 0.66 (0.38-1.15) |  |
| 9 | 0.71 (0.47-1.07) |  | 0.61 (0.36-1.04) |  |
| 10 (most advantaged; reference) | 1.00 |  | 1.00 |  |
| Veterans’ status (veteran vs non-veteran) | 1.41 (1.02-1.95) | 0.040 | 1.15 (0.75-1.78) | 0.518 |
| Healthcare card status (holder vs non-holder) | 0.76 (0.63-0.92) | 0.005 | 0.84 (0.67-1.07) | 0.166 |
| Smoking status |  |  |  |  |
| Non-smoker (reference) | 1.00 | 0.240^α^ | 1.00 | 0.149^β^ |
| Previous smoker | 1.08 (0.89-1.31) |  | 1.08 (0.86-1.37) |  |
| Smoker | 0.76 (0.49-1.17) |  | 0.65 (0.38-1.09) |  |
| **Laboratory measurements** |  |  |  |  |
| eGFR category |  |  |  |  |
| 45-59 (reference) | 1.00 | 0.223^α^ | 1.00 | 0.568^β^ |
| 30-44 | 1.03 (0.83-1.27) |  | 1.05 (0.81-1.36) |  |
| 15-29 | 1.22 (0.96-1.56) |  | 1.20 (0.89-1.60) |  |
| <15 | 1.38 (0.89-2.16) |  | 1.30 (0.75-2.25) |  |
| Serum potassium (per 0.1 mmol/L increase)* | 1.05 (1.02-1.08) | <0.001 | 1.06 (1.02-1.09) | 0.001 |
| **Comorbid conditions** |  |  |  |  |
| Atrial fibrillation (yes vs no) | 1.08 (0.88-1.33) | 0.468 | 1.13 (0.87-1.47) | 0.366 |
| Cardiovascular disease (yes vs no) | 1.03 (0.85-1.25) | 0.744 | 0.89 (0.66-1.19) | 0.433 |
| Diabetes (yes vs no) | 0.95 (0.79-1.14) | 0.598 | 0.97 (0.78-1.21) | 0.802 |
| Heart failure (yes vs no) | 1.13 (0.94-1.36) | 0.206 | 1.21 (0.91-1.60) | 0.184 |
| Left ventricular hypertrophy (yes vs no) | 1.84 (0.92-3.71) | 0.087 | 1.96 (0.87-4.43) | 0.104 |
| Stroke (yes vs no) | 1.04 (0.80-1.36) | 0.757 | 1.11 (0.80-1.55) | 0.523 |

OR=odds ration; CI=confidence interval; ARIA=Accessibility and Remoteness Index of Australia; SEIFA=socio-economic indexes for areas; eGFR=estimated glomerular filtration rate; ^α^Global p-value testing for difference in ORs across the categorical variable in the univariate model; ^β^Global p-value testing for difference in ORs across the categorical variable in the multivariable model; ^Due to small numbers in the “very remote” group, this group was combined with the “remote” group; *Serum potassium level at the time of the hyperkalemia event
