# Supplementary material for: Hyperkalemia and renin-angiotensin aldosterone system inhibitor therapy in chronic kidney disease: A general practice-based, observational study
Source: PLoS One. 2019 Mar 7;14(3):e0213192. doi: 10.1371/journal.pone.0213192 (PMC6405190; doi:10.1371/journal.pone.0213192)
Supplement: S1 Fig — (DOCX) [file pone.0213192.s004.docx]

**Supporting information**

**S1 Fig: Overview of the study design**

**
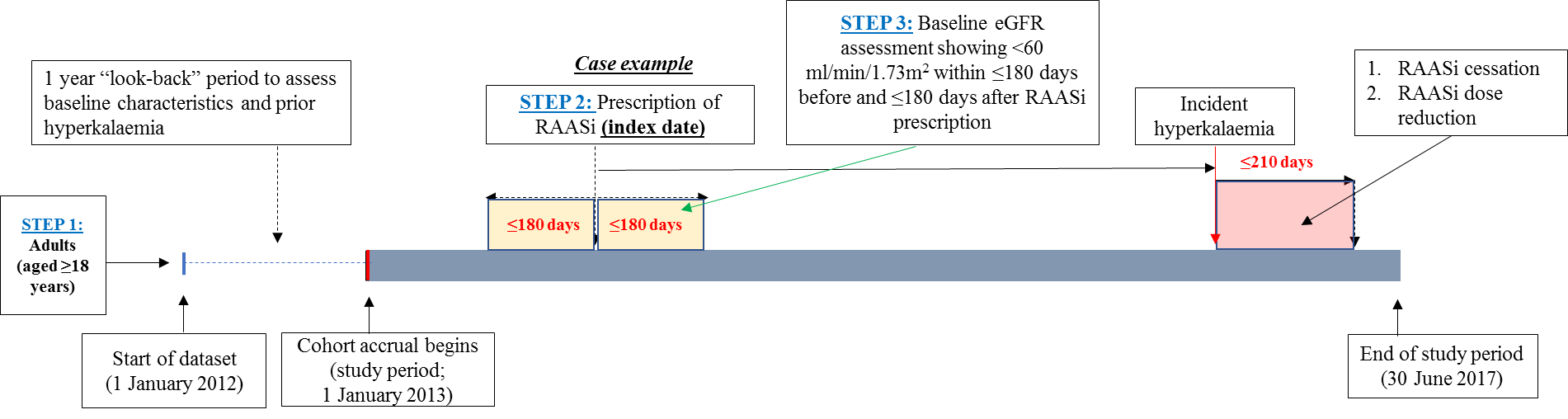
**

CKD=chronic kidney disease; RAASi=renin angiotensin aldosterone system inhibitor; eGFR=estimated glomerular filtration rate
