# Supplementary material for: Hyperkalemia and renin-angiotensin aldosterone system inhibitor therapy in chronic kidney disease: A general practice-based, observational study
Source: PLoS One. 2019 Mar 7;14(3):e0213192. doi: 10.1371/journal.pone.0213192 (PMC6405190; doi:10.1371/journal.pone.0213192)
Supplement: S2 Fig — (DOCX) [file pone.0213192.s005.docx]

**Supporting information**

**S2 Fig: Sensitivity analysis using a baseline CKD ascertainment period defined as 1 year prior to or 90 days after the index date**


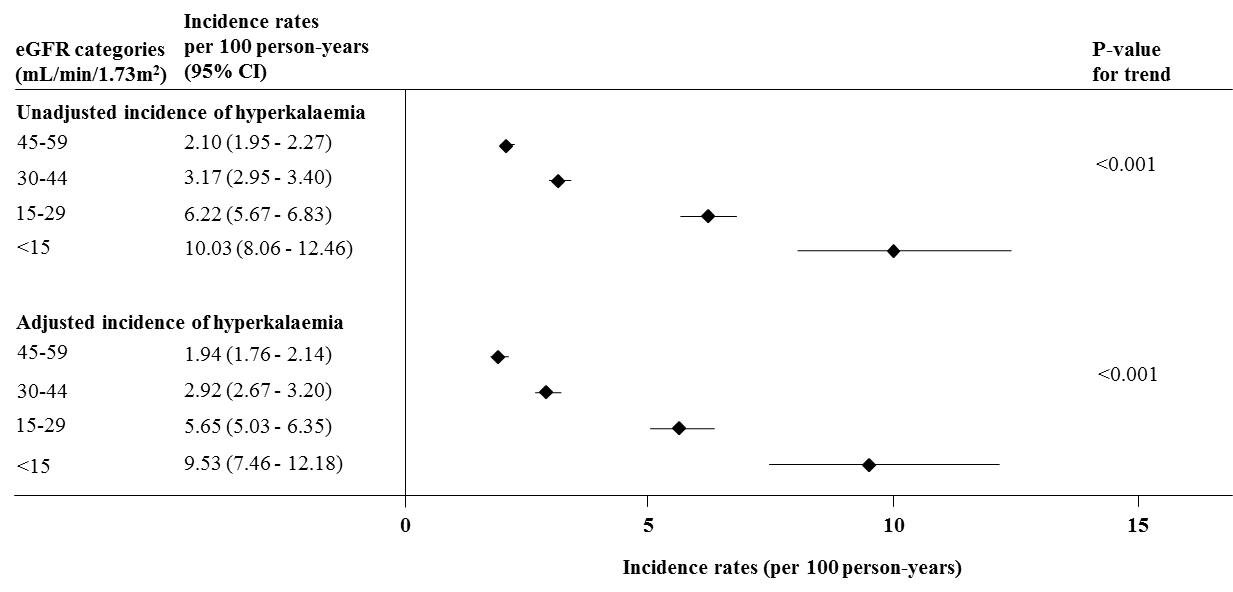


eGFR=estimated glomerular filtration rate; CI=confidence interval; adjusted for sociodemographic information (sex, age, indigenous status, region of residence, SEIFA [Socio-Economic Indexes for Areas], veteran status and healthcare card status), smoking status and comorbid conditions (atrial fibrillation, cardiovascular disease, stroke, heart failure, left ventricular hypertrophy and diabetes.
