# Supplementary material for: Hyperkalemia and renin-angiotensin aldosterone system inhibitor therapy in chronic kidney disease: A general practice-based, observational study
Source: PLoS One. 2019 Mar 7;14(3):e0213192. doi: 10.1371/journal.pone.0213192 (PMC6405190; doi:10.1371/journal.pone.0213192)
Supplement: S4 Fig — (DOCX) [file pone.0213192.s007.docx]

**Supporting information**

**S4 Fig: Unadjusted incidence rate of hyperkalemia according to serum potassium testing frequency and eGFR category**


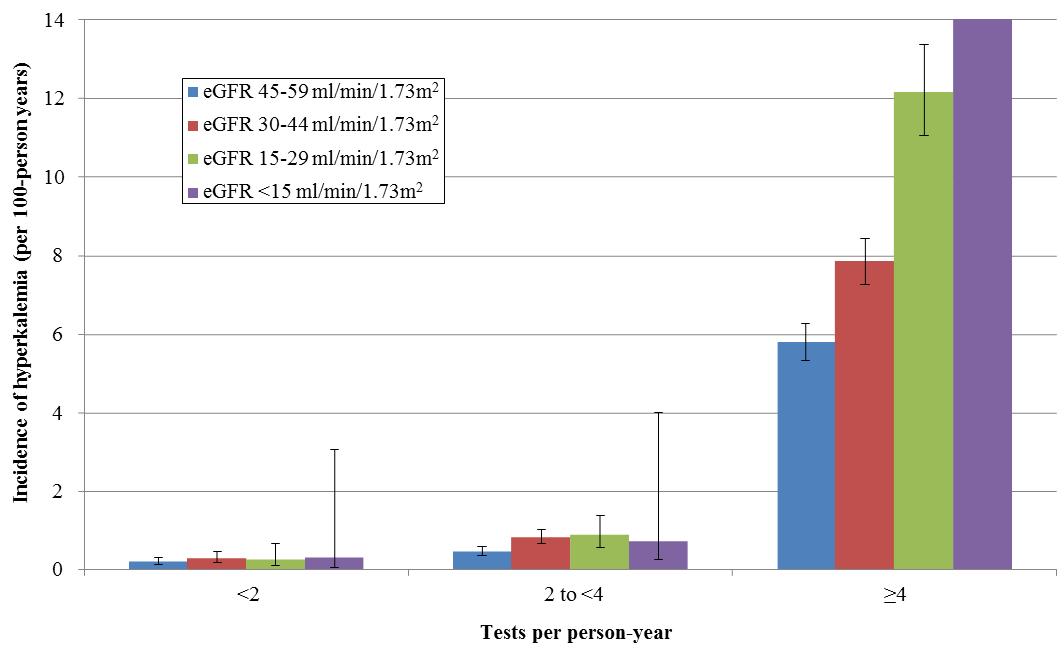


eGFR=estimated glomerular filtration rate
